# Cytotoxicity evaluation, antibacterial effect, and degree of conversion of QAM-containing adhesives

**DOI:** 10.1590/1807-3107bor-2024.vol38.0001

**Published:** 2024-01-05

**Authors:** Isadora Martini GARCIA, Tatiana Féres ASSAD-LOSS, Luis Felipe Jochinms SCHNEIDER, Fabrício Mezzomo COLLARES, Larissa Maria Assad CAVALCANTE, Mônica Almeida TOSTES

**Affiliations:** (a) University of Maryland School of Dentistry, Department of General Dentistry, Baltimore, MD, USA.; (b) Universidade Federal Fluminense – UFF, School of Dentistry, Graduate Program in Dentistry, Federal Fluminense University, Niterói, RJ, Brazil.; (c) Universidade Federal do Rio Grande do Sul - UFRGS, School of Dentistry, Laboratory of Dental Materials, Federal University of Rio Grande do Sul, Porto Alegre, RS, Brazil.

**Keywords:** Dental Bonding, Dental Caries, Dental Materials, Quaternary Ammonium Compounds, Anti-Bacterial Agents

## Abstract

The aim of this study was to evaluate the influence of adding quaternary ammonium methacrylates (QAMs) to experimental adhesives by assessing the degree of conversion (DC), cytotoxicity against keratinocytes and fibroblasts, and antibacterial activity against biofilm formation. Two QAMs were added to an experimental adhesive: dimethylaminododecyl methacrylate bromododecane (DMADDM) or dimethylaminododecyl methacrylate bromohexadecane (DMAHDM) at three concentrations each: 1, 2.5, and 5 wt.%. Experimental adhesive without QAMs (control group) and commercially available Transbond XT Primer (3M Unitek, Monrovia, California, USA) were used for comparisons. The adhesives were tested for DC, cytotoxicity against keratinocytes and fibroblasts, and antibacterial activity against biofilm formation. DC, cytotoxicity against fibroblasts, and antibacterial activity were analyzed using one-way ANOVA and Tukey’s multiple comparisons. Cytotoxicity against keratinocytes was evaluated using the Kruskal Wallis and Dunn’s post-hoc (α = 5%) tests. Transbond showed lower DC as compared to 5% DMAHDM, 1% DMADDM, and 5% DMADDM (p < 0.05). However, all groups presented proper DC when compared to commercial adhesives in the literature. In the evaluation of cytotoxicity against keratinocytes, Transbond induced higher viability than 2.5 wt.% groups (p < 0.05). Against fibroblasts, Transbond induced higher viability as compared to 5 wt.% groups (p < 0.05). DMAHDM at 5 wt.% reduced biofilm formation when compared to all the other groups (p < 0.05). Despite their cytotoxic effect against keratinocytes, gingival fibroblasts showed higher viability. DMAHDM at 5 wt.% decreased *Streptococcus mutans* viability. The incorporation of DMAHDM at 5 wt.% may be a strategy for reducing the development of white spot lesions.

## Introduction

Restorative dental materials with antimicrobial properties have been developed to prevent recurrent caries.^1^ More recently, resins with antimicrobial activity have also been proposed to avoid white spot lesions (WSLs) in patients wearing orthodontic appliances.^2-4^ Patients treated with orthodontic aligners are at a lower risk for developing WSLs thanks to shorter treatment length and easier oral hygiene.^5^ However, excess bonding material for attachments left on the tooth surface may represent a risk for the development of WSLs.^6^.Even though the prior application of adhesive resins (also called “primer” in orthodontics) has been reported as a step that could be set aside during bracket bonding,^7^ a particular indication for these materials could be the promotion of antimicrobial activity to protect enamel against demineralization.^2^ In this context, the incorporation of antimicrobial agents into adhesive resins could be considered a suitable approach because they come into direct contact with enamel surfaces.^8^ Furthermore, the use of restorative materials or orthodontic bonding of accessories containing therapeutic agents may improve resistance to mechanical and acidic challenges in the oral environment.^9^

Among antimicrobial agents, quaternary ammonium methacrylates (QAMs) alone,^3,10-14^ associated with silver nanoparticles,^1,9,15,16^ or with nanoparticles of amorphous calcium phosphate^4,17,18^have commanded attention because they promote bacterial cell lysis via direct contact^14^ and can be copolymerized within the resin matrix.^11^ Two QAMs – DMADDM^1,3,12,15,19-21^and DMAHDM^4,11,13,19,20,22^– have been investigated at different concentrations. Although many studies have chosen to test such monomers at concentrations from 3 to 10 wt.% with adequate results in mechanical tests and antibacterial activity,^19,20,23^ cytotoxicity may be a limiting factor ^10^. The release of antibacterial agents into the surrounding site has several disadvantages: a decrease in the mechanical properties of the parental resin-based material over time, short-term effectiveness, and possible cytotoxicity.^24^ QAMs with different aliphatic chains have been previously tested against human fibroblasts and odontoblasts.^25^ The materials were tested directly in contact with the cells and eluates from polymerized samples. The tests were performed in an MTT assay with serial dilution. Currently, the use of a test with better predictive power than the MTT, such as the sulforhodamine B (SRB) assay, has been advocated.^26^ Furthermore, other cells such as keratinocytes could be evaluated, given their possible contact with resins in the region close to the gingiva.

DMADDM at 1.5 wt.%, 3 wt.%, and 5 wt.% had already been added to a commercial adhesive, and a remarkable reduction in the metabolic activity of microorganisms was observed at 3 wt.%.^3^ In another study, when DMADDM was tested at 2.5 wt.% and 5 wt.%, incorporation of 5 wt.% showed higher antibacterial activity against mature biofilms.^21^ The antibacterial activity of DMADDM in a commercial adhesive was dependent on the incubation period.^12^ DMADDM at 2.5 wt.% was only effective in reducing bacterial viability after a longer incubation period (48 h and 72 h). At 5 wt.%, the reduction of bacterial activity was effective even after a 16-hour incubation period. Even after this longer incubation period, bacterial activity in the 2.5 wt.% group was lower than in the 5 wt.% group.^12^

When DMAHDM was tested at 0.75 wt.%, 1.5 wt.%, 2.25 wt.%, and 3 wt.% in two incubation periods (48 h and 72 h), the metabolic activity of biofilm decreased at all concentrations when compared to the control group.^13^ The higher the concentration, the greater the antibacterial activity.^11^ Currently, better antibacterial activity and maintenance of physicochemical properties have been observed when DMAHDM is added at 5 wt.% to resin materials, such as resin composites.^17^

In this context, the longer the carbon chain and the concentration of QAMs, the higher the antimicrobial activity. However, these two factors can also impair resin-based physical properties.^19,20^ Besides, chemical properties such as the DC of parental resin-based materials should be considered. Efficient polymerization is a critical parameter to ensure optimal physical properties and reduce susceptibility to cytotoxic effects.^27^ Although DMADDM and DMAHDM have been analyzed in dental materials, few studies have directly compared them.^10,19,20^ Both QAMs are very similar, with one methacrylate functional group and one ammonium functional group, with a difference of four carbons in the aliphatic chain. This study aimed to evaluate the DC, cytotoxic effects, and antibacterial activity of two QAMs (DMADDM and DMAHDM) at three different concentrations (1, 2.5, or 5 wt.%). The null hypothesis to be tested is that the addition of QAMs does not affect the DC, cytotoxicity, and antibacterial activity of orthodontic adhesives.

## Methodology

### Experimental adhesive formulation

**Figure 1:**
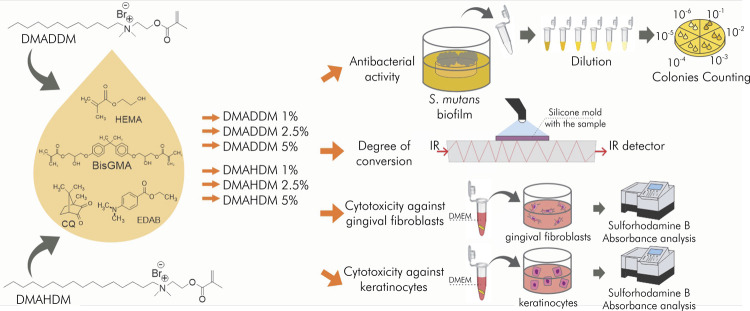
Illustration of the study design. The chemical structures of DMADDM and DMAHDM are indicated, evidencing longer alkyl chain (16 carbons) of DMAHDM as compared to DMADDM (12 carbons) and the presence of the quaternary ammonium group in both structures. The base resin was formulated with BisGMA, HEMA, and photoinitiators. DMADDM and DMAHDM were incorporated at 1, 2.5, and 5 wt.% into this experimental resin. All materials were tested for antibacterial activity, degree of conversion, cytotoxicity against gingival fibroblasts, and cytotoxicity against keratinocytes.

The adhesive formulation^28,29^ was the mixture of bisphenol-A-glycidyldimetacrylate (Bis-GMA, Esstech, Essington, USA) and 2-hydroxyethyl methacrylate (HEMA, Esstech, Essington, USA) at a 50:40 wt.% ratio. As the photoinitiator/co-initiator system, 0.5 mol% of camphorquinone (Esstech Inc., Essington, USA) and 1 mol% of EDMAB (Sigma-Aldrich, St Louis, USA) were added to this base resin. QAMs were synthesized according to previous studies.^10,15,19,30^ The chemical structures of both QAMs are indicated in Figure 1 together with an illustration of the study design. DMADDM and DMAHDM were added at 1, 2.5, and 5 wt.% from the total organic matrix weight. The groups were categorized as follows:

- Transbond – commercially available Transbond XT Primer adhesive (3M Unitek, Monrovia, USA);
- Control – experimental adhesive without QAMs;
- 1% DMADDM – experimental adhesive with 1 wt.% of DMADDM;
- 2.5% DMADDM – experimental adhesive with 2.5 wt.% of DMADDM;
- 5% DMADDM – experimental adhesive with 5 wt.% of DMADDM;
- 1% DMAHDM – experimental adhesive with 1 wt.% of DMAHDM;
- 2.5% DMAHDM – experimental adhesive with 2.5 wt.% of DMAHDM;
- 5% DMAHDM – experimental adhesive with 5 wt.% of DMAHDM.

### Degree of conversion

The samples of adhesives (n = 3^31^) were placed on the attenuated total reflectance device of a Fourier-transform infrared (FTIR) spectrometer (Alpha-P; Bruker Optics, Ettlingen, Germany). Each sample was photoactivated for 40 s using a light-emitting diode (LED, Radii Cal, SDI, Bayswater, Victoria, Australia) with 1,200 mW/cm^2^. This light-curing unit was used to photoactivate the adhesives throughout the study. Photoactivation was standardized at 1 mm between the tip of the light-curing unit and the top of the samples. Spectra from FTIR analysis were recorded before and immediately after the photoactivation of each sample with 32 scans and a resolution of 4 cm^1^. The DC was calculated based on the height of the peak at 1,638 cm^1^ (carbon-carbon double bonds of aliphatic chain) and the height of the peak at 1,608 cm^1^ peak (carbon-carbon double bonds of aromatic chain) using the following formula:


$$DC(\%)=100\times \frac{\frac{\text{ peak height of cured aliphatic }C=C)}{(\text{ peak height of cured aromatic }C=C}}{\frac{\text{ peak height of uncured aliphatic }C=C)}{\text{ (peak height of uncured aromatic }C=C}}$$

## Cytotoxicity against keratinocytes

For cytotoxicity evaluation of experimental orthodontic adhesives against keratinocytes, disc-shaped samples (n = 5, 1.0 mm thickness × 4.0 mm diameter^32,33^) were prepared after 20 s of photoactivation on each side. The samples were stored in distilled water at 37°C for 24 h, dried with absorbent paper, and sterilized using hydrogen peroxide plasma at 58 % for 48 min at 56°C. The samples were stored in 1 mL of Dulbecco’s Modified Eagle Medium (DMEM, Sigma-Aldrich Chemical, St. Louis, USA) for 24 h at 37°C for eluate preparation. Human keratinocytes (HaCaT, CLS Cell Lines Service GmbH, Eppelheim, Germany) were placed at 5 × 10^3^ per well in 96-well plates to be treated with 100 μL of eluate from each sample. After 72 h of incubation at 37°C with 5% CO_2_, cells were fixed on the bottom of the wells by trichloroacetic acid solution at 10 vol.%. The plates were incubated for 1 h at 4°C, washed with running water for 30 s, and dried at room temperature. The cells were stained with an SRB sodium salt (SRB, Sigma-Aldrich Chemical) at 0.4 vol.%. After 30 min at room temperature, the plates were washed with acetic acid 1 vol.% and dried at room temperature. Trizma solution (10 mM, 100 μL) was added to each well to dissolve the dye before absorbance assessment. After 1 h, absorbance was analyzed at 560 nm. A negative control composed of cells without contact with eluates was used as 100%. The results were expressed in percentages of cell viability.

### Cytotoxicity against gingival fibroblasts

Gingival fibroblasts were collected after a periodontal procedure and informed consent was obtained from the patient after approval by the local Research Ethics Committee (process nº 1.739.340). The patient donated the tissue after its removal according to therapeutic indication after signing the informed consent form. Firstly, the cells were grown in flasks containing nutrient medium. The medium was composed of DMEM with 10% fetal bovine serum, 5 mM of Hepes, 3.7 g sodium bicarbonate, 100 U/mL of penicillin, and 100 mg/mL of streptomycin. The cells were incubated at 37°C with 5% CO_2_. The cells were evaluated daily with the aid of an inverted-phase microscope, and the medium was changed every 2 to 3 days. After cell growth, new samples of adhesives were prepared (n = 5, 1.0 mm thickness × 4.0 mm diameter^32,33^) with photoactivation for 20 s on each side. The samples were stored in distilled water at 37°C for 24 h, dried with absorbent paper, and sterilized using hydrogen peroxide plasma at 58 % for 48 min at 56°C. The samples used to produce the eluates were prepared according to the description above for the evaluation of cytotoxicity against keratinocytes. The cells were seeded (5 × 10^3^ per well) in 96-well plates and treated with 100 μL of eluate from each sample. The contact period between the eluates and the cells was 72 h. Thereafter, the cells were treated according to the description above using the SRB method. The absorbance results were also normalized against the cell viability in wells with cells without contact with the eluates and expressed as percentage.

### Antibacterial activity against biofilm formation

To evaluate antibacterial activity, disc-shaped samples of the orthodontic adhesives were prepared (n = 3, 1.0 mm thickness × 4.0 mm diameter^16,34^) with photoactivation for 20 s on each side. The samples were stored in distilled water at 37°C for 24 h and dried with absorbent paper. The samples were attached onto the lid of a 48-well plate, and this set was sterilized using hydrogen peroxide plasma at 58 % for 48 min at 56°C. In each well of the 48-well plate, 900 μL of brain heart infusion (BHI) broth was added. The BHI broth contained 1 wt.% of sucrose and 100 μL of a suspension of an overnight broth culture of S. mutans (NCTC 10449) at 10^6^ CFU/mL. The 48-well plate containing the samples was incubated for 24 h at 37°C. After that, the samples were detached from the lid, vortexed for 1 min in 1 mL of sterile saline solution (0.9 wt.%) in an Eppendorf tube. The solution was serially diluted up to 10^6^ mL of saline solution. Then, two drops of 25 μL were plated on Petri dishes with BHI agar. The plates were incubated for 48 h at 37°C.^16,34^

### Statistical analysis

The data were analyzed using SigmaPlot software (version 12.0, Systat Software, San Jose, USA). Data distribution was evaluated by the Shapiro-Wilk test. DC, cytotoxicity against fibroblasts, and antibacterial activity were analyzed using one-way ANOVA and Tukey’s multiple comparisons. Cytotoxicity against keratinocytes was evaluated using the Kruskal Wallis and Dunn’s post-hoc tests. A significance level of 5% was used for all tests.

## RESULTS

**Figure 2:**
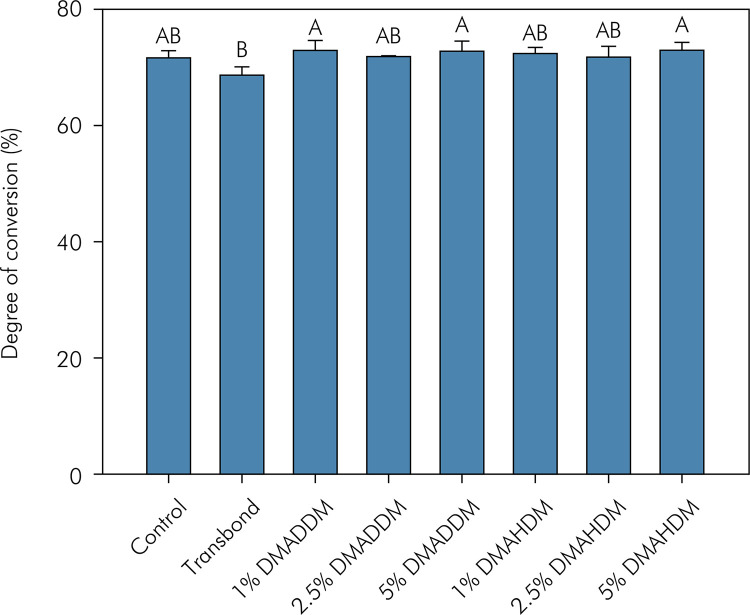
Mean and standard deviation of degree of conversion (%) of the orthodontic adhesives. Different capital letters indicate statistically significant differences among groups (p > 0.05).

The DC of the adhesives is displayed in Figure 2. Transbond showed lower DC than 5% DMAHDM, 1% DMADDM, and 5% DMADDM (p < 0.05). There were no statistically significant differences among the other groups (p > 0.05).

The results for keratinocyte viability are presented in Table 1. There were statistically significant differences among the experimental adhesive groups (p < 0.05). Transbond showed no difference for the control group, neither for the adhesives containing 1% or 5% of QAMs (p > 0.05). There was a statistically significant difference between Transbond and both groups with 2.5 wt.% of QAMs (p < 0.05), without statistical difference between 2.5% DMAHDM and 2.5% DMAHDM (p > 0.05).

**Table 1:** Keratinocyte viability after contact with eluates from polymerized samples.

Group	Median (50th percentile)	25th percentile	75th percentile	Statistical result
Control	23.20	19.48	26.84	AB
Transbond	98.72	96.78	99.44	A
1% DMADDM	17.53	15.29	29.93	AB
2.5% DMADDM	13.03	10.30	16.51	B
5% DMADDM	23.50	16.21	26.20	AB
1% DMAHDM	15.72	14.01	30.13	AB
2.5% DMAHDM	12.61	11.76	13.82	B
5% DMAHDM	15.42	14.67	20.81	AB

Different capital letters indicate a statistically significant difference among groups (p < 0.05).

**Figure 3:**
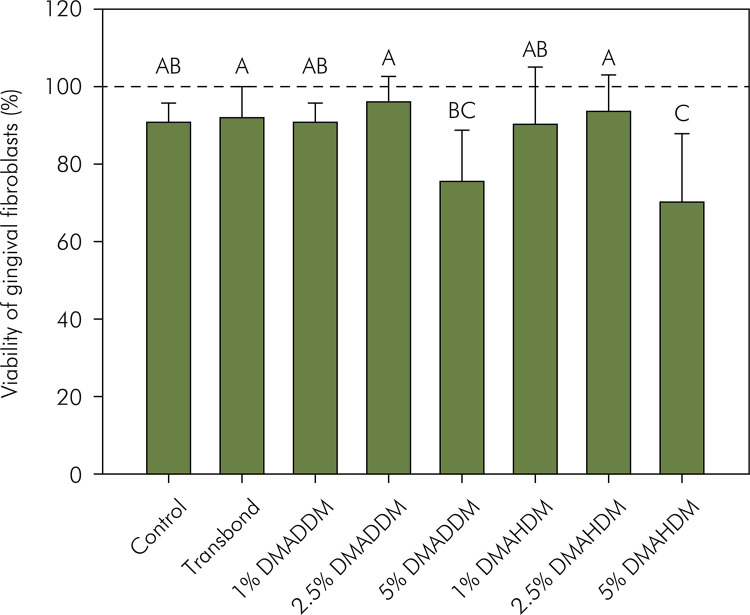
Mean and standard deviation of gingival fibroblast viability (%). Different capital letters indicate statistically significant differences among groups (p < 0.05).

The results for fibroblast viability are displayed in Figure 3. The addition of 5 wt.% of DMAHDM reduced cell viability when compared to the control group and Transbond (p < 0.05). The group with 5 wt.% of DMADDM showed lower cell viability than Transbond (p < 0.05), without statistical difference for the control group (p > 0.05). There was no difference between the groups containing 5 wt.% of QAMs (p > 0.05).

**Figure 4:**
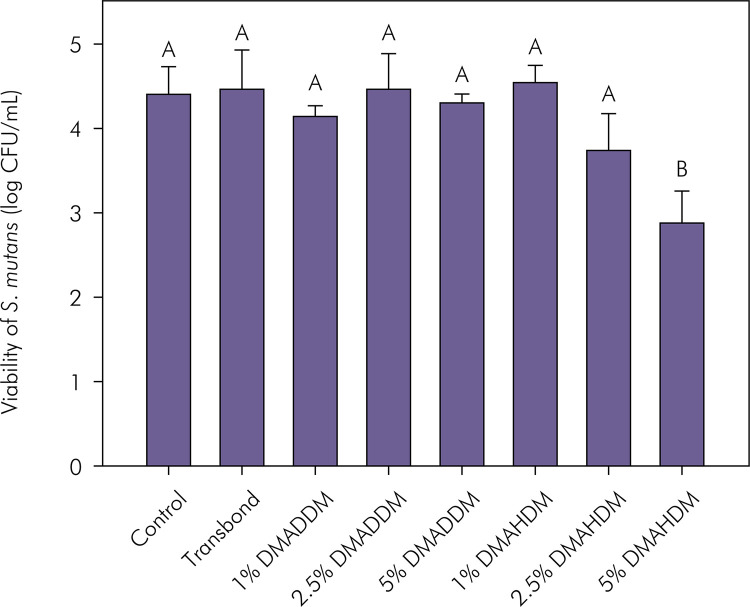
Mean and standard deviation of antibacterial activity against biofilm formation after contact with the orthodontic adhesives. Different capital letters indicate statistically significant differences among groups (p <0.05).

Figure 4 indicates the log CFU/mL of viable S. mutans on biofilm formed on the polymerized samples. The group with 5 wt.% of DMAHDM induced more than 1 log reduction in relation to the control and Transbond groups (p < 0.05). There was a statistical difference between the groups, with lower bacterial viability for the group with 5 wt.% of DMAHDM as compared to all the other adhesives (p < 0.05), without statistically significant difference among the other groups (p > 0.05).

## Discussion

In the present study, two types of QAMs were explored in orthodontic adhesives at different concentrations. The formulated materials, together with a commercial control (Transbond), were analyzed for chemical and biological properties. There was no difference in DC among the groups. However, biological properties differed among groups in cytotoxicity and antibacterial activity evaluations. Therefore, the null hypothesis was partially rejected.

High DC is essential for achieving proper mechanical properties and hydrolytic stability of dental adhesives.^31^ The incorporation of monomers with different densities and quantity of methacrylate groups affects the polymerization kinetics and the DC of adhesives.^31^In this way, the DC of resins doped with QAMs should be analyzed to help elucidate the polymerization behavior of the developed materials. Transbond showed lower DC than the groups with 5 wt.% of QAMs and 1% DMADDM (p < 0.05). It was not clear why there was statistically significant difference among these four groups. However, we could observe that, regardless of the statistical outcome, all groups showed proper DC when compared to the literature, achieving more than 50 % of DC.^35,36^

Considering ISO 10993-5, all experimental adhesives showed cytotoxicity against keratinocytes because all these groups presented values above 70%.^37^ Previous studies have reported higher viability when DMAHDM at 5 wt.% was used in sealants^23^and DMADDM at 2.5 and 5 wt.% was used in adhesive resins.^12^ By contrast, another study that tested solutions containing DMADDM showed significant cytotoxic effects on human buccal epithelial cells.^38^ All experimental adhesives showed cell viability lower than 70%, while Transbond presented higher values, suggesting that other components and concentrations could be involved in this process and lead to cytotoxicity.

The cytotoxicity of monomers was ranked as follows: BisGMA > UDMA > TEGDMA > HEMA.^39^Transbond is composed of TEGDMA, BisGMA, and photoinitiator systems. Therefore, the eluted HEMA from our adhesives may have affected the viability of keratinocytes. Moreover, differences in network formation between experimental adhesives and Transbond could differ in quantity and type of components release. The risk of adverse effects on other tissues is emphasized because these materials can release a great variety of toxic compounds, which may diffuse across biological membranes and, therefore, exert adverse effects either locally or beyond the oral cavity.^40^ Although BisGMA has been identified as one of the most cytotoxic monomers,^39,40^ it is widely used in dental resins. DMADDM, which is 20 times less toxic than BisGMA, could probably also be acceptable for clinical use.^41^

Cytotoxicity tests are performed to determine how a specific cell type could be affected by an experimental material. Of note, the in vitro test applied may have overestimated the cytotoxic effect of the materials. In bracket bonding or cavity restoration, only a thin layer of adhesive is used, which contrasts with the thickness of the samples and with the storage conditions used. However, this strategy is employed to observe the behavior of materials when subjected to major challenges, using a high concentration of eluates (the eluates were not diluted) and high exposure (72 hours). Although the experiments with in vitro cells cannot reproduce in vivo conditions, they are widely used as a simplified method of investigation and restrict the number of experimental variables.^37^

The evaluation of cytotoxicity against fibroblasts indicated a higher percentage of cell viability. All experimental groups showed values over 70%, which is in line with previous analyses using fibroblasts.^42^ We decided to test the possible cytotoxicity of the adhesives against fibroblasts because of the original site of these cells (gingiva) and its possible proximity to the orthodontic adhesive in a clinical situation. Furthermore, ISO recommends the use of fibroblasts to analyze the cytotoxicity potential of materials.^37^ The findings reported herein for fibroblast viability suggest that the adhesives with DMAHDM or DMADDM up to 5 wt.% do not present cytotoxicity.

The antibacterial activity was assessed via quantification of viable S. mutans on the biofilm formed on the polymerized samples. The group containing DMAHDM at 5 wt.% showed antibacterial activity with a reduction of more than 1 log CFU/mL compared to the control groups. A previous study found that an increase in DMADDM mass fraction reduces the metabolic activity of biofilms.^43^ However, in the present study, bacterial viability did not change for DMADDM groups after diluting and plating the bacterial content. This difference may be associated with the antibacterial tests applied. The MTT assay is a colorimetric test to assess the metabolic activity of cells. Despite being widely used, it has been suggested that tests that analyze cell viability rather than cell metabolism be applied. As with tests based solely on morphological changes of cell membranes, tests based on metabolism generally have less predictive power.^44^ For this reason, we chose to perform tests that quantify cell viability both in the analysis of cytotoxicity (SRB method) and in the study of antibacterial activity (colony-forming units assay).

The higher antibacterial effect of DMAHDM as compared to DMADDM at the same concentration (5 wt.%) is attributed to the longer alkyl chain of DMAHDM. As mentioned earlier, DMAHDM has 16 carbons in the aliphatic chain, while DMADDM has 12. A previous study has analyzed the effect of QAMs with three, six, nine, twelve, sixteen, and eighteen carbons in the aliphatic chain against S. mutans biofilm. The authors observed a decreased minimum inhibitory concentration (MIC) by increasing the aliphatic chain of QAM. Moreover, there was decreased biofilm formation on polymerized samples with longer-chain methacrylates.^25^.Another study evaluated this effect against microcosm biofilm derived from dental plaque. From three to sixteen carbons, there was also improved antibacterial property.^25^ The rationale for these outcomes is the higher hydrophobicity and improved capacity of DMAHDM to penetrate the hydrophobic microbial membrane compared to QAMs with shorter aliphatic chains.^25^

As a limitation of this study, we did not quantify the released compounds. Further research could analyze this effect after soaking the materials in solutions for different periods. This analysis could contribute to a deeper understanding of the biological effects of materials. The formulated orthodontic adhesives present a comonomer blend that could also be used for dentin bonding. Therefore, further research could also be performed by analyzing adhesives with 5 wt.% of DMAHDM to improve antibacterial activity and assist in preventing recurrent caries around resin-based restorations.^2^

## Conclusion

The addition of QAMs did not change DC when compared to the experimental control group, and some QAM groups had higher DC than Transbond. Even though there was decreased keratinocyte viability, gingival fibroblasts showed proper viability. Furthermore, DMAHDM at 5 wt.% decreased *Streptococcus mutans* viability. The incorporation of DMAHDM at 5 wt.% may be a strategy for reducing the development of WSLs.
